# Periostin Augments Vascular Smooth Muscle Cell Calcification via β-Catenin Signaling

**DOI:** 10.3390/biom12081157

**Published:** 2022-08-21

**Authors:** Ioana Alesutan, Laura A. Henze, Beate Boehme, Trang T. D. Luong, Daniel Zickler, Burkert Pieske, Kai-Uwe Eckardt, Andreas Pasch, Jakob Voelkl

**Affiliations:** 1 Institute for Physiology and Pathophysiology, Johannes Kepler University Linz, Altenberger Strasse 69, 4040 Linz, Austria; 2 Department of Internal Medicine and Cardiology, Campus Virchow-Klinikum, Charité—Universitätsmedizin Berlin, Augustenburger Platz 1, 13353 Berlin, Germany; 3 Department of Nephrology and Medical Intensive Care, Charité—Universitätsmedizin Berlin, Augustenburger Platz 1, 13353 Berlin, Germany; 4 DZHK (German Centre for Cardiovascular Research), Partner Site Berlin, 13347 Berlin, Germany; 5 Department of Internal Medicine and Cardiology, German Heart Center Berlin (DHZB), Augustenburger Platz 1, 13353 Berlin, Germany; 6 Berlin Institute of Health (BIH), Anna-Louisa-Karsch 2, 10178 Berlin, Germany; 7 Calciscon AG, Aarbergstrasse 46, 2503 Biel, Switzerland

**Keywords:** vascular calcification, vascular smooth muscle cells, phosphate, periostin, chronic kidney disease, β-catenin

## Abstract

Medial vascular calcification is common in chronic kidney disease (CKD) and is closely linked to hyperphosphatemia. Vascular smooth muscle cells (VSMCs) can take up pro-calcific properties and actively augment vascular calcification. Various pro-inflammatory mediators are able to promote VSMC calcification. In this study, we investigated the effects and mechanisms of periostin, a matricellular signaling protein, in calcifying human VSMCs and human serum samples. As a result, periostin induced the mRNA expression of pro-calcific markers in VSMCs. Furthermore, periostin augmented the effects of β-glycerophosphate on the expression of pro-calcific markers and aggravated the calcification of VSMCs. A periostin treatment was associated with an increased β-catenin abundance as well as the expression of target genes. The pro-calcific effects of periostin were ameliorated by WNT/β-catenin pathway inhibitors. Moreover, a co-treatment with an integrin αvβ3-blocking antibody blunted the pro-calcific effects of periostin. The silencing of periostin reduced the effects of β-glycerophosphate on the expression of pro-calcific markers and the calcification of VSMCs. Elevated serum periostin levels were observed in hemodialysis patients compared with healthy controls. These observations identified periostin as an augmentative factor in VSMC calcification. The pro-calcific effects of periostin involve integrin αvβ3 and the activation of the WNT/β-catenin pathway. Thus, the inhibition of periostin may be beneficial to reduce the burden of vascular calcification in CKD patients.

## 1. Introduction

Medial vascular calcification is a multifaceted and regulated process frequently observed in chronic kidney disease (CKD) and is associated with cardiovascular outcomes [1]. Hyperphosphatemia in CKD has been associated with pro-inflammatory and pro-calcific effects in the vasculature [2]. These effects are partly mediated by vascular smooth muscle cells (VSMCs), which can undergo a phenotypical alteration and change the vascular environment to favor the deposition of calcium and phosphate [2]. The development of pro-calcific VSMCs is orchestrated by complex signaling pathways [3]. A variety of pro-inflammatory signaling molecules have been shown to modulate pro-calcific pathways in VSMCs and augment their calcification [4]. Interference with pro-inflammatory mediators may prevent the development of vascular calcification [5].

Periostin is a matricellular protein involved in bone remodeling, cardiovascular differentiation, tumor growth and metastasis as well as inflammation [6]. To mediate its effects, periostin binds to integrins, especially ανβ3 [7]. In addition to integrins, discoidin domain receptor-1 was shown to be a putative periostin receptor [8]. Periostin is expressed in osteocytes and is considered to play an important role in bone homeostasis [9], but it is also expressed in various other tissues [10]. Periostin expression can be upregulated in various pathological conditions [11] and has been associated with atherosclerotic valve disease [12]. In vascular tissue, the upregulation of periostin was observed in VSMCs after a balloon injury in rats [13]. The upregulation of periostin in neointima formation occurs in conjunction with the upregulation of αvβ3 and αvβ5 integrins [14]. Periostin-deficient mice showed a reduced development of atherosclerotic lesions and inflammatory responses with an ApoE-deficient background [15]. VSMC migration is stimulated by periostin, an effect involving an interaction with integrins ανβ3 and ανβ5 [16]. Hypoxia, a known stimulator of VSMC calcification [17], upregulates periostin expression in pulmonary arterial VSMCs [18]. An increased periostin expression has been observed in the calcifying vasculature of uremic rats [19] and a role of periostin in VSMC calcification has been suggested [20]. In addition to the vasculature, periostin has been linked to renal fibrosis in CKD [21]. After a unilateral ureteral obstruction, the renal periostin expression is upregulated and a periostin deficiency ameliorates renal fibrosis [22]. On the other hand, periostin improved renal ischemia/reperfusion injuries in mice [23]. Urinary periostin was further suggested as a marker of a renal injury in type 2 diabetes mellitus [24]. Furthermore, serum periostin has been investigated as a biomarker [11]. As examples, higher serum periostin levels were observed in patients with an ossification of the posterior longitudinal ligament [25] or coronary artery calcifications [20].

Thus, periostin may be an important factor in VSMC calcification during CKD. Therefore, in this study we investigated the function of periostin and its mechanisms during VSMC calcification.

## 2. Materials and Methods

### 2.1. Cell Culture

Primary human aortic VSMCs (HAoSMCs), commercially obtained from Fisher Scientific (Vienna, Austria) and Sigma Aldrich (Vienna, Austria), were routinely cultured as previously described [5,26]. The cells were used in experiments up to passage 12. The HAoSMCs were treated for 24 h (mRNA expression and protein abundance) or 7 days (ALP activity) with 100 ng/mL recombinant human periostin (stock in PBS, R&D Systems, Abingdon, UK) [20,27], 2 mM β-glycerophosphate (Sigma Aldrich, Vienna, Austria) [28], 1 nM LGK974 (stock in DMSO, Cayman Chemical, Ann Arbor, MI, USA) [29], 10 µM XAV939 (stock in DMSO, Cayman Chemical, Ann Arbor, MI, USA) [30], 10 µM PRI-724 (stock in DMSO, Selleckchem, Planegg, Germany) [31] and 1 µg/mL anti-integrin αvβ3 antibody (ab78289, Abcam, Cambridge, UK) [32] or mouse IgG (R&D Systems, Abingdon, UK). The HAoSMCs were treated with equal amounts of the vehicle as a control. HAoSMCs were transfected with 10 nM POSTN (ID: s20888) or negative control (ID: 4390843) siRNA using a siPORT amine transfection reagent (all from Fisher Scientific, Vienna, Austria) [33]. For the calcification experiments, the HAoSMCs were treated for 11 days with 10 mM β-glycerophosphate and 1.5 mM CaCl_2_ (Sigma Aldrich, Vienna, Austria) as a calcification medium [34]. For the long-term treatments, fresh media with agents were added every two to three days.

### 2.2. RNA Isolation and RT-PCR

The total RNA was isolated using a Trizol reagent (Fisher Scientific, Vienna, Austria). The synthesis of cDNA was performed with Superscript III Reverse Transcriptase and oligo(dT)_12–18_ primers (Fisher Scientific, Vienna, Austria) [26] as well as RT-PCR using iQ Sybr Green Supermix (Bio-Rad Laboratories, Vienna, Austria) and the following human primers (Fisher Scientific, Vienna, Austria) [28,35,36,37]:

*ALPL* fw: GGGACTGGTACTCAGACAACG;
*ALPL* rev: GTAGGCGATGTCCTTACAGCC;
*CBFA1* fw: GCCTTCCACTCTCAGTAAGAAGA;
*CBFA1* rev: GCCTGGGGTCTGAAAAAGGG;
*GAPDH* fw: GAGTCAACGGATTTGGTCGT;
*GAPDH* rev: GACAAGCTTCCCGTTCTCAG;
*MMP2* fw: TACAGGATCATTGGCTACACACC;
*MMP2* rev: GGTCACATCGCTCCAGACT;
*MSX2* fw: TGCAGAGCGTGCAGAGTTC;
*MSX2* rev: GGCAGCATAGGTTTTGCAGC;
*PIT1* fw: GGAAGGGCTTGATTGACGTG;
*PIT1* rev: CAGAACCAAACATAGCACTGACT;
*POSTN* fw: GCTATTCTGACGCCTCAAAACT;
*POSTN* rev: AGCCTCATTACTCGGTGCAAA;
*WNT3A* fw: AGCTACCCGATCTGGTGGTC;
*WNT3A* rev: CAAACTCGATGTCCTCGCTAC;
*WNT7A* fw: CTGTGGCTGCGACAAAGAGAA;
*WNT7A* rev: GCCGTGGCACTTACATTCC.

The relative mRNA expression was calculated using the 2^−ΔΔCt^ method with GAPDH as the housekeeping gene, normalized to the control group.

### 2.3. Protein Isolation and Western Blotting

Total proteins were isolated using an ice-cold Pierce IP lysis buffer (Fisher Scientific, Vienna, Austria) supplemented with complete protease and a phosphatase inhibitor cocktail (Fisher Scientific, Vienna, Austria) [36,38]. The protein concentration was determined by a Bradford assay (Bio-Rad Laboratories, Vienna, Austria). Equal amounts of protein were incubated in Roti-Load1 Buffer (Carl Roth, Karlsruhe, Germany) at 100 °C for 10 min and then separated on SDS-PAGE gels and transferred to PVDF membranes. The membranes were incubated with primary rabbit anti-β-catenin (1:1000, #8480, Cell Signaling, Frankfurt am Main, Germany) or rabbit anti-GAPDH (1:1000, #2118, Cell Signaling, Frankfurt am Main, Germany) antibodies at 4 °C overnight and with a secondary anti-rabbit HRP-conjugated antibody (1:1000, Cell Signaling, Frankfurt am Main, Germany) at RT for 1 h. The membranes were stripped in a stripping buffer (Fisher Scientific, Vienna, Austria) at RT. Bands were detected with an ECL detection reagent (Fisher Scientific, Vienna, Austria) and quantified using ImageJ software ((NIH, MD, USA, 1.52n). The data were shown as the ratio of the total protein to GAPDH, normalized to the control group [38,39].

### 2.4. ALP Activity Assay

ALP activity in the cell lysates was determined by using a colorimetric ALP assay kit (Abcam, Cambridge, UK). The protein concentration was determined by a Bradford assay (Bio-Rad Laboratories, Vienna, Austria). The data were shown normalized to the total protein concentration and to the control group [38,40].

### 2.5. Determination of Calcification

The HAoSMCs were fixed with 4% paraformaldehyde/PBS and stained with 2% Alizarin Red (pH 4.5) [39]. The calcification was shown as a red stain. To determine the calcium content, the HAoSMCs were decalcified in 0.6 M HCl at 4 °C overnight and then the total proteins were isolated using a 0.1 M NaOH/0.1% SDS buffer and quantified by a Bradford assay (Bio-Rad Laboratories, Vienna, Austria). The calcium content was measured with a QuantiChrom Calcium assay kit (BioAssay Systems, Hayward, CA, USA). The data were shown normalized to the total protein concentration [39,41].

### 2.6. Human Samples

A patient cohort of healthy controls and CKD patients with a measurement of serum calcification propensity determined by a one-half maximal transition time (T50) of in vitro transformation from primary to secondary calciprotein particles using a Nephelostar Plus nephelometer (BMG Labtech, Ortenberg, Germany) was previously described in detail [34]. The serum periostin levels were determined using a human Periostin DuoSet ELISA kit (#DY3548B, R&D Systems, Abingdon, UK) and a DuoSet Ancillary Reagent Kit 2 (R&D Systems, Abingdon, UK).

### 2.7. Statistics

The data were shown as scatter dot plots and arithmetic means ± SEM. The normalized data were presented as arbitrary units (a.u.). *N* represented the number of independent experiments performed. The normality was determined using a Shapiro–Wilk test. Non-normal datasets were transformed (log, sqrt or rec) prior to the statistical testing to provide normality. For two groups, statistical testing was performed using an unpaired two-tailed *t*-test or a Mann–Whitney U test. For the multiple group comparison, statistical testing was performed using a one-way ANOVA with a Tukey (homoscedastic data) or a Games–Howell (heteroscedastic data) post hoc test and a Kruskal–Wallis test with a Steel–Dwass post hoc test (non-normal data). For the correlation analysis, a Spearman correlation was performed. A *p* < 0.05 was considered to be significant.

## 3. Results

**Figure 1:**
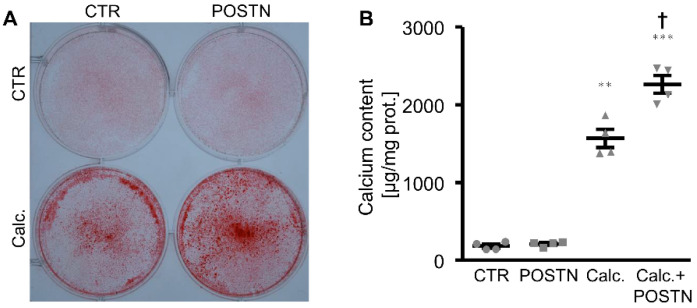
Periostin aggravates phosphate-induced calcification of HAoSMCs. (**A**) Alizarin Red staining in HAoSMCs treated with a control (CTR) or calcification medium (Calc.) without or with additional treatment with recombinant human periostin (POSTN). Calcified areas: red staining. (**B**) Calcium content in HAoSMCs treated with a control (CTR) or calcification medium (Calc.) without or with additional treatment with recombinant human periostin (POSTN). ** *p* < 0.01; *** *p* < 0.001 (significant difference versus CTR group); † *p* < 0.05 (significant difference versus Calc. group).

**Figure 2:**
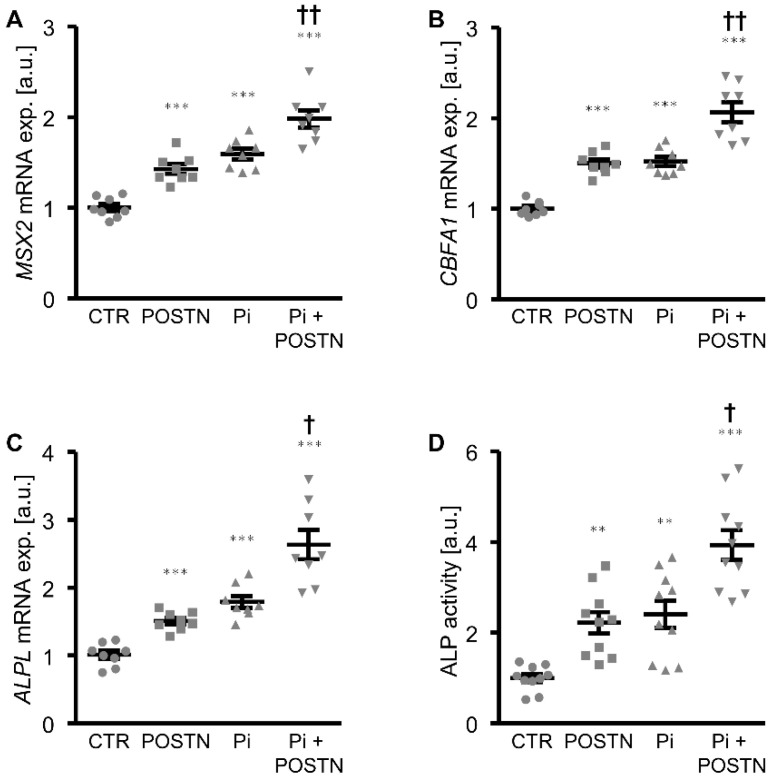
Periostin increases osteogenic signaling and augments osteoinduction promoted by phosphate in HAoSMCs. (**A**–**C**) Relative mRNA expression of *MSX2* (**A**), *CBFA1* (**B**) and *ALPL* (**C**) in HAoSMCs treated with control (CTR) or β-glycerophosphate (Pi) without or with additional treatment with recombinant human periostin (POSTN). (**D**) Normalized ALP activity in HAoSMCs treated with control (CTR) or β-glycerophosphate (Pi) without or with additional treatment with recombinant human periostin (POSTN). ** *p* < 0.01; *** *p* < 0.001 (significant difference versus CTR group); † *p* < 0.05; †† *p* < 0.01 (significant difference versus Pi group).

The first experiments investigated whether periostin directly impacted the phosphate-induced calcification of VSMCs in vitro. As shown by Alizarin Red staining and the measurement of the calcium content, the calcification of the HAoSMCs induced by the calcification medium was significantly aggravated in the presence of the recombinant human periostin protein (Figure 1). Moreover, the periostin treatment alone was sufficient to significantly upregulate the osteogenic marker expression and activity in the HAoSMCs, as shown by an increased mRNA expression of *MSX2*, *CBFA1* and *ALPL* (Figure 2A–C) as well as the ALP activity (Figure 2D). The effects of β-glycerophosphate on the osteogenic marker expression and ALP activity in the HAoSMCs were augmented by an additional treatment with periostin. Thus, periostin triggered pro-calcific effects and aggravated the calcification of the HAoSMCs.

**Figure 3:**
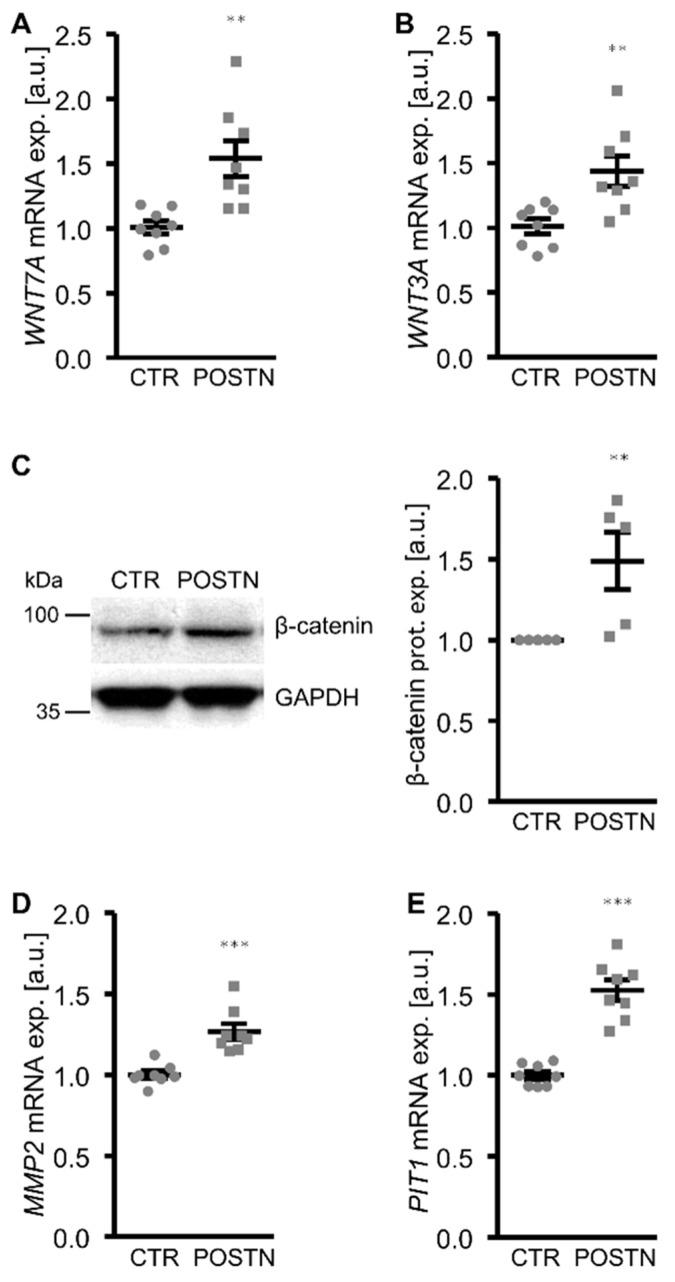
Periostin increases WNT/β-catenin signaling in HAoSMCs. (**A**,**B**) Relative mRNA expression of *WNT7A* (**A**) and *WNT3A* (**B**) in HAoSMCs treated with control (CTR) or recombinant human periostin (POSTN). (**C**) Representative Western blots and normalized β-catenin protein expression in HAoSMCs treated with control (CTR) or recombinant human periostin (POSTN). (**D**,**E**) Relative mRNA expression of *MMP2* (**D**) and *PIT1* (**E**) in HAoSMCs treated with control (CTR) or recombinant human periostin (POSTN). ** *p* < 0.01; *** *p* < 0.001 (significant difference versus CTR group).

**Figure 4:**
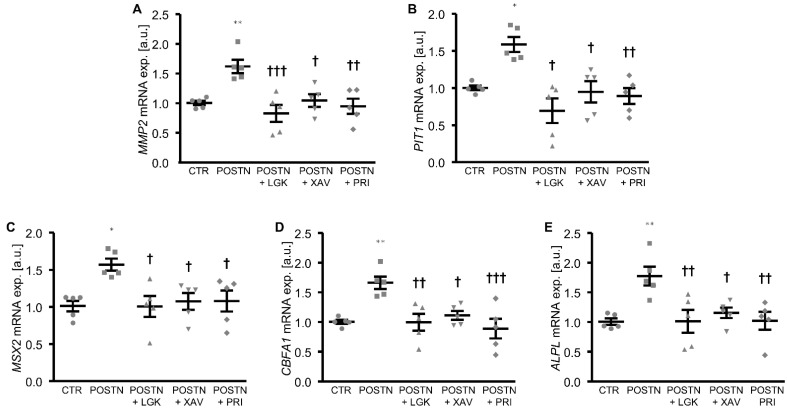
Inhibition of the WNT/β-catenin pathway suppresses periostin-induced osteogenic signaling in HAoSMCs. (**A**–**E**) Relative mRNA expression of *MMP2* (**A**), *PIT1* (**B**), *MSX2* (**C**), *CBFA1* (**D**) and *ALPL* (**E**) in HAoSMCs treated with control (CTR) or recombinant human periostin (POSTN) without or with additional treatment with the WNT/β-catenin pathway inhibitors LGK974 (LGK), XAV939 (XAV) or PRI-724 (PRI). * *p* < 0.05; ** *p* < 0.01 (significant difference versus CTR group); † *p* < 0.05; †† *p* < 0.01; ††† *p* < 0.001 (significant difference versus POSTN group).

To elucidate the mechanisms involved in the pro-calcific effects of periostin in the HAoSMCs, the potential role of the WNT/β-catenin pathway as a mediator was investigated. The addition of periostin to the cell culture medium significantly upregulated the *WNT7A* and *WNT3A* mRNA expression and β-catenin protein abundance in the HAoSMCs (Figure 3A–C). Furthermore, the mRNA expression of *MMP2* and *PIT1*, β-catenin target genes with key roles in VSMC calcification, was significantly higher in the periostin-exposed HAoSMCs than in the control HAoSMCs (Figure 3D,E). An additional treatment of HAoSMCs with WNT/β-catenin pathway inhibitors (LGK974, which inhibits WNT post-translational acylation; XAV939, which stimulates β-catenin degradation; or PRI-724, which blocks the interaction of the coactivator CBP with β-catenin) all significantly suppressed periostin-induced *MMP2*, *PIT1* and the osteogenic marker mRNA expression (Figure 4). Taken together, the pro-calcific effects of periostin in the HAoSMCs were mediated, at least partly, by the WNT/β-catenin signaling pathway.

**Figure 5:**
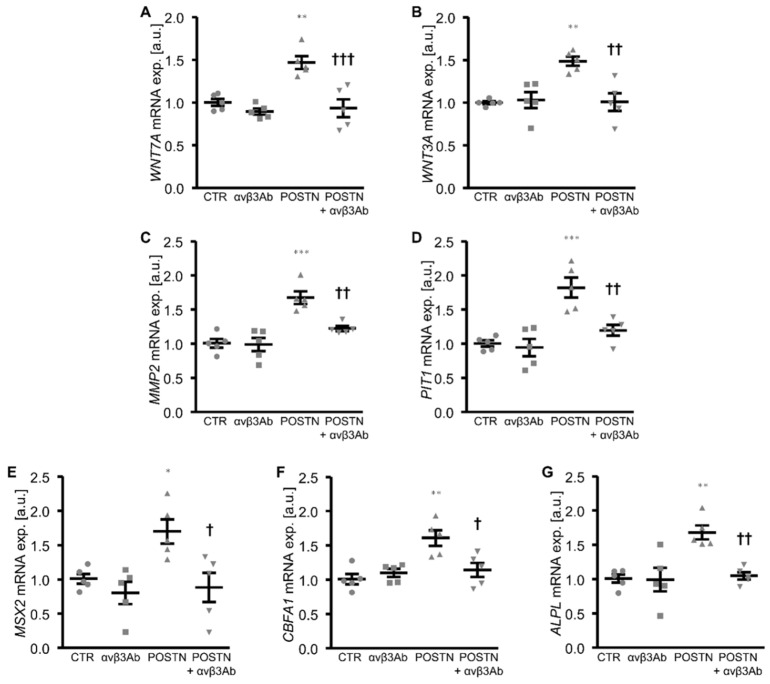
Treatment with integrin αvβ3 antibody blunts periostin-induced WNT/β-catenin and osteogenic signaling in HAoSMCs. (**A**–**G**) Relative mRNA expression of *WNT7A* (**A**), *WNT3A* (**B**), *MMP2* (**C**), *PIT1* (**D**), *MSX2* (**E**), *CBFA1* (**F**) and *ALPL* (**G**) in HAoSMCs treated with control (CTR) or recombinant human periostin (POSTN) and with mouse IgG as control or integrin αvβ3 antibody (αvβ3Ab). * *p* < 0.05; ** *p* < 0.01; *** *p* < 0.001 (significant difference versus CTR group); † *p* < 0.05; †† *p* < 0.01; ††† *p* < 0.001 (significant difference versus POSTN group).

Further experiments explored the role of integrin αvβ3, a receptor of periostin, in the pro-calcific effects of periostin in the HAoSMCs. To this end, the HAoSMCs were exposed to periostin in the presence and absence of an integrin αvβ3-blocking antibody. As a result, the increased mRNA expression of *WNT7A*, *WNT3A*, *MMP2*, *PIT1* and osteogenic markers upon periostin exposure was significantly inhibited in the presence of the integrin αvβ3 antibody (Figure 5), suggesting that the periostin-induced activation of WNT/β-catenin signaling and its pro-calcific effects in the HAoSMCs involved integrin αvβ3.

**Figure 6:**
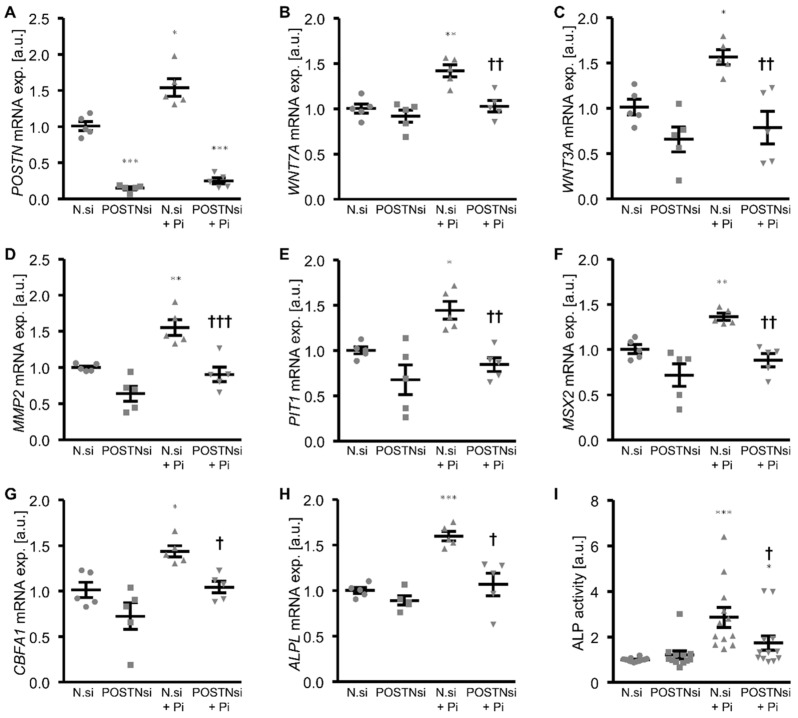
Silencing of periostin ameliorates phosphate-induced WNT/β-catenin and osteogenic signaling in HAoSMCs. (**A**–**H**) Relative mRNA expression of *POSTN* (**A**), *WNT7A* (**B**), *WNT3A* (**C**), *MMP2* (**D**), *PIT1* (**E**), *MSX2* (**F**), *CBFA1* (**G**) and *ALPL* (**H**) in HAoSMCs transfected with negative control siRNA (N.si) or POSTN siRNA (POSTNsi) and treated with control or β-glycerophosphate (Pi). (**I**) Normalized ALP activity in HAoSMCs transfected with negative control siRNA (N.si) or POSTN siRNA (POSTNsi) and treated with control or β-glycerophosphate (Pi). * *p* < 0.05; ** *p* < 0.01; *** *p* < 0.001 (significant difference versus N.si group); † *p* < 0.05; †† *p* < 0.01; ††† *p* < 0.001 (significant difference versus N.si + Pi group).

**Figure 7:**
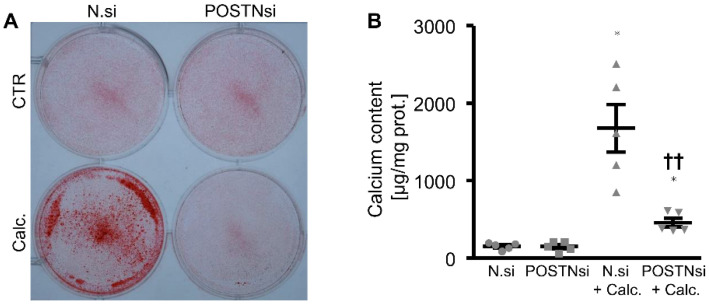
Silencing of periostin reduces phosphate-induced calcification of HAoSMCs. (**A**) Alizarin Red staining in HAoSMCs transfected with negative control siRNA (N.si) or POSTN siRNA (POSTNsi) and treated with control (CTR) or calcification medium (Calc.). Calcified areas: red staining. (**B**) Calcium content in HAoSMCs transfected with negative control siRNA (N.si) or POSTN siRNA (POSTNsi) and treated with control (CTR) or calcification medium (Calc.). * *p* < 0.05 (significant difference versus N.si group); †† *p* < 0.01 (significant difference versus N.si + Calc. group).

To further explore whether endogenous periostin played a role in the phosphate-induced osteogenic phenotypical switch and calcification of the HAoSMCs, the periostin expression was suppressed by silencing using small interfering RNA (siRNA). As shown in Figure 6A, the *POSTN* mRNA expression was significantly reduced in the POSTN siRNA-transfected HAoSMCs than in the negative control siRNA-transfected HAoSMCs. The phosphate treatment significantly upregulated the *POSTN* mRNA expression in the negative control siRNA-transfected HAoSMCs. Moreover, the silencing of periostin significantly blunted the phosphate-induced *WNT7A*, *WNT3A*, *MMP2*, *PIT1* and osteogenic marker mRNA expression (Figure 6B–H) as well as the ALP activity (Figure 6I) in the HAoSMCs. In accordance, a knockdown of periostin significantly reduced the phosphate-induced calcification of the HAoSMCs (Figure 7). Thus, endogenous periostin participated in phosphate-induced osteogenic signaling and calcification of the HAoSMCs.

**Figure 8:**
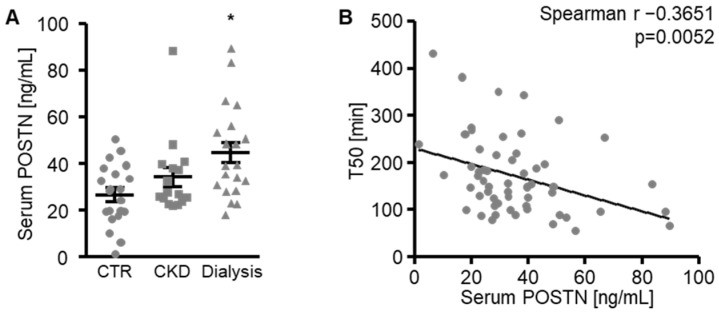
Serum periostin levels are increased in hemodialysis patients. (**A**) Serum periostin (POSTN) levels in healthy volunteers (CTR), patients with known CKD (CKD) and hemodialysis patients (Dialysis). * *p* < 0.05 (significant difference versus CTR group). (**B**) Correlation between serum periostin (POSTN) concentrations and serum calcification propensity measured as calciprotein particle maturation time (T50). The *p*-value is indicated in the figure.

Further pilot experiments explored a possible association between the serum periostin levels and serum calcification propensity (T50). The periostin levels were determined in the serum from healthy volunteers, patients with known CKD and hemodialysis patients [34]. As shown in Figure 8A, the serum periostin levels were not significantly changed in the CKD patients (*p* = 0.4375), but were significantly higher in the hemodialysis patients compared with the healthy volunteers. The serum periostin levels inversely correlated with the T50 (Figure 8B), suggesting that increased periostin levels were associated with calcification propensity.

## 4. Discussion

The current results further support a critical role of periostin in vascular calcification. The effects of periostin involve integrin αvβ3 activation and the WNT/β-catenin pathway. In our model, exposure to β-glycerophosphate upregulated the periostin expression in human aortic VSMCs. Although the mechanisms and effects differ between calcifying VSMCs and osteoblasts, an upregulated periostin expression was observed in both cell types during VSMC calcification and osteoblast differentiation [42]. Furthermore, an upregulated periostin expression was observed in calcifying dermal fibroblasts [43], calcified human aortic valves [44], atherosclerotic plaques [45] and left ventricular tissues of rats with chronic renal failure [46]. In rats, the left ventricular periostin expression was increased by angiotensin 2 or vasopressin, known stimulators of vascular calcification [3,46,47]. Periostin expression in the kidney has also been reported in CKD [21]. Periostin supplementation is able to augment calcification whilst the silencing of endogenous periostin can ameliorate calcification in human aortic VSMCs. This suggests a functional relevance of periostin in vascular calcification during CKD. Notably, the effects of periostin may differ during embryonic development [48].

The WNT/β-catenin pathway is apparently required for the pro-calcific effects of periostin in VSMCs. Periostin has previously been implicated as an activator of the WNT/β-catenin pathway [49,50]. WNT3A and WNT7A stimulate β-catenin [51] and are associated with vascular calcification [52]. β-catenin has emerged as a critical pathway augmenting VSMC calcification [2,3,53] and regulates the expression of osteogenic transcription factor CBFA1/RUNX2 [29], matrix metalloproteinase 2 (MMP2) [54] and the sodium-dependent phosphate cotransporter PIT1 [55]. An increased β-catenin expression was reported during the osteogenesis of mesenchymal stem cells [50]. In accordance, β-catenin and the osteogenic differentiation of mesenchymal stem cells were impaired by a periostin knockdown [56]. Pharmacological interference with the WNT/β-catenin pathway blocks the stimulating effects of periostin on the mRNA expression of *CBFA1*, *MMP2* and *PIT1*. Thus, the pro-calcific effects of periostin are partly mediated by β-catenin.

The pro-calcific effects of periostin further involved integrin αvβ3, as suggested by the inhibition of the periostin-induced osteogenic marker expression in VSMCs by the αvβ3-blocking antibody. Integrin αvβ3 antibodies have previously been used to block the effects of periostin or other agonists [16,57,58,59]. In accordance, integrin αvβ3 contributes to the stimulating effect of periostin on VSMC migration [16]. The expression of αvβ3 in human aortic atheromas is associated with the plaque burden and inflammation [60]. In valvular interstitial cells, integrin αvβ3 is associated with pro-calcific effects [61]. In VSMCs, calcification is promoted by fibronectin, which can also bind to integrin αvβ3 [62,63]. However, integrin αvβ3 also binds osteopontin, which may act as an inhibitor of VSMC calcification [64,65]. The effects of osteopontin may be mediated by directly blocking hydroxyapatite growth [65]. Although the current observations suggest a role of integrin αvβ3 in the pro-calcific effects of periostin, the involvement of other receptors or pathways cannot be ruled out. Furthermore, the current study was limited in the readouts with in vitro observations with primary human vascular smooth muscle cells. The artificial conditions and primary cells used in the cell culture experiments could affect the observed results and may not necessarily exactly reflect the alterations in the vasculature, especially during uremic conditions. Clearly, further studies in vivo are required to verify the effects of periostin and the therapeutic potential of its inhibition in the vasculature.

The uremic environment with hyperphosphatemia induces a pro-inflammatory state, which is considered to be a key mechanism for accelerated calcification in CKD [2]. Multiple inflammatory mediators have been linked to the pro-calcific environment in CKD [2,4,36]. Periostin has been connected with vascular and renal inflammatory processes [15,21,22,24] and, therefore, may be an important aspect in the complex signaling pathways of vascular calcification. The current observations are indicative of a functional role of periostin in CKD-associated vascular calcification. Although our pilot study was observational and has limitations, the observation of serum periostin levels in the hemodialysis patients and the association of periostin with the serum calcification propensity support a putative relevance of periostin in CKD. The serum calcification propensity has emerged as a novel cardiovascular risk factor [66] and has been linked to coronary artery calcification severity and progression in CKD patients [67]. Furthermore, the serum calcification propensity has been associated with mortality in CKD [68], patients with ischemic heart failure [69] and the general population [70]. However, a causal link between periostin and the calcification propensity cannot be made based on the current observations. The serum calcification propensity reflects the sum of pro- and anti-calcific mechanisms in the serum at a given time, but does not necessarily reflect the calcification burden per se [67]. Further studies are required to confirm and extend the association of periostin with vascular calcification in CKD patients.

In conclusion, the current observations described a pro-calcific effect of periostin in human VSMCs. This effect apparently involved the activation of integrin αvβ3 and β-catenin to induce the upregulation of the osteogenic marker expression in VSMCs. The silencing of periostin ameliorated the VSMC calcification, suggesting a putative therapeutic benefit of periostin inhibition in CKD; however, further studies in vivo and in CKD patients are warranted.

## Data Availability

The data presented in this study are available from the corresponding author on reasonable request.
